# Supplementary material for: Characterization of the MG828507 lncRNA Located Upstream of the FLT1 Gene as an Etiology for Pre-Eclampsia
Source: J Clin Med. 2022 Aug 7;11(15):4603. doi: 10.3390/jcm11154603 (PMC9369602; doi:10.3390/jcm11154603)
Supplement: Supplementary file 1 [file jcm-11-04603-s001.zip › jcm-1731250-supplementary.pdf]

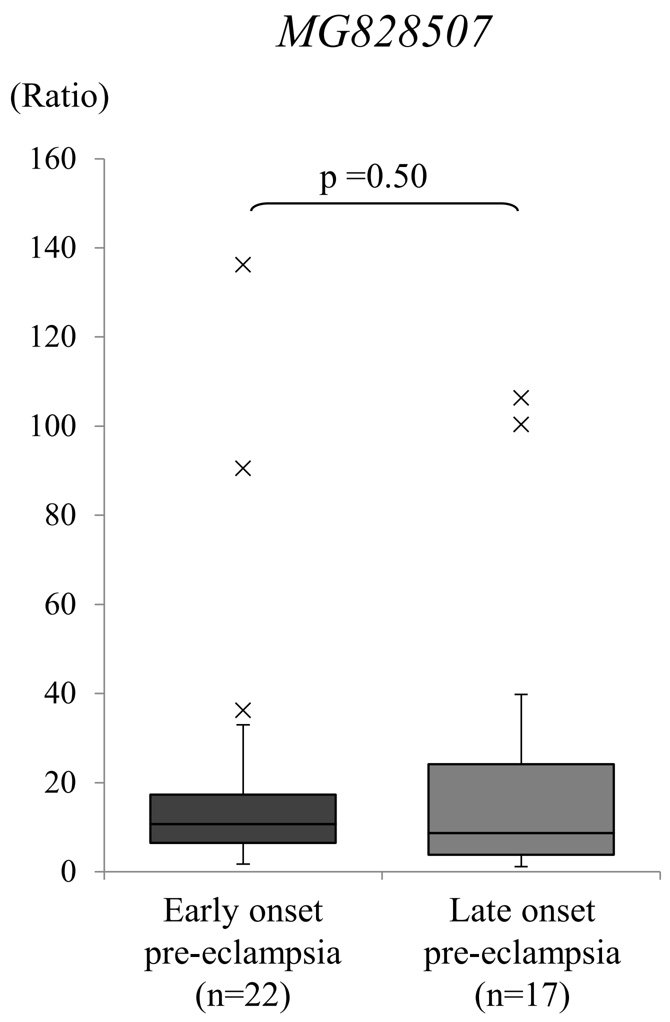

Supplementary Figure S1: Correlations between *MG828507* expression and disease onset

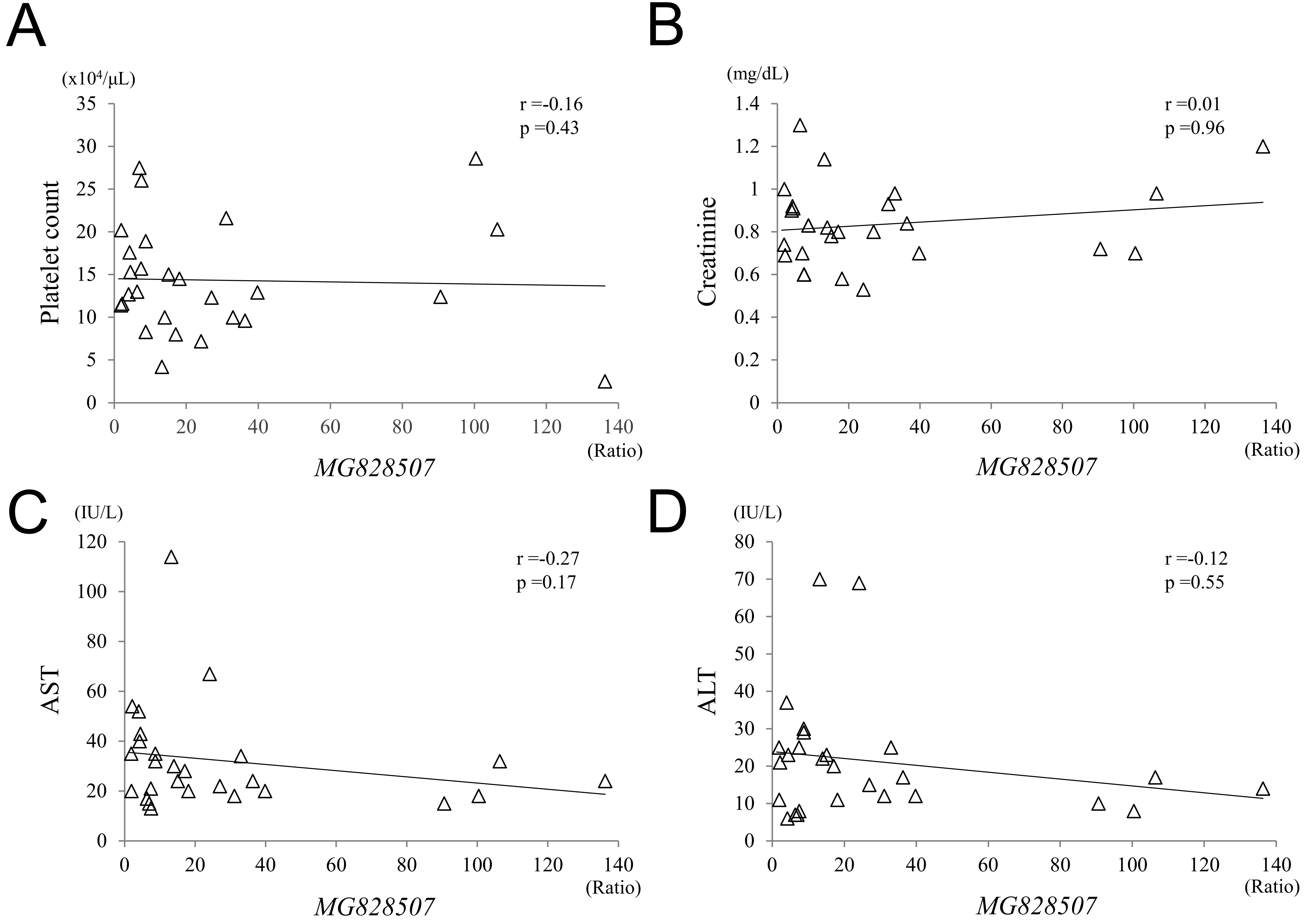

Supplementary Figure S2: Correlations between *MG828507* expression and clinical parameters
